# Association Between Premenstrual Disorders and Quality of Life: A Cross-Sectional Study of 8311 women in Sweden

**DOI:** 10.1192/j.eurpsy.2025.573

**Published:** 2025-08-26

**Authors:** Q. Wang, D. Lu

**Affiliations:** 1 Karolinska Institutet, Stockholm, Sweden; 2 Institute of Environmental Medicine, Karolinska Institutet, Stockholm, Sweden

## Abstract

**Introduction:**

Premenstrual disorders (PMDs), including premenstrual syndrome (PMS) and premenstrual dysphoric disorder (PMDD), manifest a range of affective, cognitive, behavioral, and physical symptoms during the late luteal phase of the menstrual cycle. Several studies have indicated that women with PMDs have a lower health-related quality of life, though findings on specific affected dimensions vary. Most of them relied on community- or clinical setting-based sampling, limiting the generalization. Furthermore, no prior study has formally assessed the association by accounting for the potential confounders. Therefore, we conducted a cross-sectional study based on the LifeGene cohort to evaluate the difference in quality of life among women with and without PMDs.

**Objectives:**

We seek to understand, to what extent and in which way PMDs may impact the quality of life of women in Sweden.

**Methods:**

We conducted a cross-sectional study of 8311 women enrolled to the LifeGene cohort during 2009-2019 in Sweden. PMDs were assessed with the modified Premenstrual Symptom Screening Tool at baseline, while data on quality of life (QoL) was obtained using the EQ-5D-3L scale (the higher the score, the worse the quality of life) from the LifeGene baseline dataset. Linear regression models were employed to assess the QoL score change associated with PMDs, as well as the separate analyses for PMDD and PMS. The models were adjusted for age, education level, country of birth, marital status, obesity, smoking, alcohol consumption, and childhood abuse.

**Results:**

At a mean age of 34.01 years (SD=9.63), 1903 (22.90%) women met the criteria for PMDs. In the crude analysis, PMDs were positively associated with a lower quality of life (mean increased QoL score: 0.37, CI: 0.32-0.42). After adjustment for demographics and potential confounders, similar results were yielded (mean increased QoL score: 0.34, CI: 0.29-0.39). Among the five indicators included in the questionnaire (mobility, self-care, activity, pain and anxiety), only self-care was not associated with PMDs. For PMD subtypes, we found a lower quality of life among women with PMDD (mean increased QoL score: 0.35, CI: 0.30-0.41) but not among women with PMS.

**Conclusions:**

Our findings suggested that women with PMDD had a lower quality of life compared to those without PMDD. While evidence based on prospective data is warranted in future, effective management is needed to improve the quality of life for women suffering PMDD.

**Disclosure of Interest:**

None Declared

